# The impact of study design and diagnostic approach in a large multi-centre ADHD study. Part 1: ADHD symptom patterns

**DOI:** 10.1186/1471-244X-11-54

**Published:** 2011-04-07

**Authors:** Ueli C Müller, Philip Asherson, Tobias Banaschewski, Jan K Buitelaar, Richard P Ebstein, Jaques Eisenberg, Michael Gill, Iris Manor, Ana Miranda, Robert D Oades, Herbert Roeyers, Aribert Rothenberger, Joseph A Sergeant, Edmund JS Sonuga-Barke, Margaret Thompson, Stephen V Faraone, Hans-Christoph Steinhausen

**Affiliations:** 1 Department of Child and Adolescent Psychiatry, University of Zurich, Switzerland; 2 Hochschule für Heilpädagogik, Zurich, Switzerland; 3 MRC Social Genetic Developmental and Psychiatry Centre, Institute of Psychiatry, London, UK; 4 Department of Child and Adolescent Psychiatry and Psychotherapy, Central Institute of Mental Health, J 5, Mannheim, Germany; 5 Department of Psychiatry, Radboud University Nijmegen Medical Center, Nijmegen, The Netherlands; 6 Department of Psychology, Hebrew University, Jerusalem, Israel; 7 Department of Psychiatry, School of Medicine, Trinity College Dublin, Dublin, Ireland; 8 Geha MHC, Petach-Tikva, Israel; 9 Department of Developmental and Educational Psychology, University of Valencia, Valencia, Spain; 10 Clinic for Child and Adolescent Psychiatry and Psychotherapy, University of Duisburg-Essen, Essen, Germany; 11 Department of Experimental Clinical and Health Psychology, Ghent University, Ghent, Belgium; 12 Department of Child and Adolescent Psychiatry, University of Göttingen, Göttingen, Germany; 13 Department of Clinical Neuropsychology, Vrije Universiteit, Amsterdam, The Netherlands; 14 School of Psychology, University of Southampton, Southampton, UK; 15 Departments of Psychiatry and of Neuroscience and Physiology, SUNY Upstate Medical University, Syracuse, NY, USA; 16 Aalborg Psychiatric Hospital, Aarhus University Hospital, Aalborg, Denmark; 17 Clinical Psychology and Epidemiology, Institute of Psychology, University of Basel, Basel, Switzerland

**Keywords:** ADHD, multi-centre study, sibling design, ADHD, informant effects, centre effects

## Abstract

**Background:**

The International Multi-centre ADHD Genetics (IMAGE) project with 11 participating centres from 7 European countries and Israel has collected a large behavioural and genetic database for present and future research. Behavioural data were collected from 1068 probands with the combined type of attention deficit/hyperactivity disorder (ADHD-CT) and 1446 'unselected' siblings. The aim was to analyse the IMAGE sample with respect to demographic features (gender, age, family status, and recruiting centres) and psychopathological characteristics (diagnostic subtype, symptom frequencies, age at symptom detection, and comorbidities). A particular focus was on the effects of the study design and the diagnostic procedure on the homogeneity of the sample in terms of symptom-based behavioural data, and potential consequences for further analyses based on these data.

**Methods:**

Diagnosis was based on the Parental Account of Childhood Symptoms (PACS) interview and the DSM-IV items of the Conners' teacher questionnaire. Demographics of the full sample and the homogeneity of a subsample (all probands) were analysed by using robust statistical procedures which were adjusted for unequal sample sizes and skewed distributions. These procedures included multi-way analyses based on trimmed means and winsorised variances as well as bootstrapping.

**Results:**

Age and proband/sibling ratios differed between participating centres. There was no significant difference in the distribution of gender between centres. There was a significant interaction between age and centre for number of inattentive, but not number of hyperactive symptoms. Higher ADHD symptom frequencies were reported by parents than teachers. The diagnostic symptoms differed from each other in their frequencies. The face-to-face interview was more sensitive than the questionnaire. The differentiation between ADHD-CT probands and unaffected siblings was mainly due to differences in hyperactive/impulsive symptoms.

**Conclusions:**

Despite a symptom-based standardized inclusion procedure according to DSM-IV criteria with defined symptom thresholds, centres may differ markedly in probands' ADHD symptom frequencies. Both the diagnostic procedure and the multi-centre design influence the behavioural characteristics of a sample and, thus, may bias statistical analyses, particularly in genetic or neurobehavioral studies.

## Background

Attention Deficit Hyperactivity Disorder ADHD is characterized by problems in allocating attention, regulating motor activity, and controlling behavioural impulses. Depending on diagnostic procedures, around 3 to 8 percent of the children worldwide are affected by ADHD [1,2]. According to dominant symptom clusters, three diagnostic subtypes of ADHD are distinguished: inattentive type (ADHD-IT), hyperactive/impulsive type (ADHD-HT), and combined type (ADHD-CT) [3].

At least half of the children with ADHD suffer from one or more comorbid disorders, of which oppositional defiant disorder, conduct disorder, anxiety disorders, and mood disorders are the most common [4-7]. Although symptoms of inattention and, even more markedly, hyperactivity and impulsivity, decline from childhood to adolescence [8], ADHD may persist completely or partially into adulthood and may constitute a risk factor for mood and anxiety disorders, substance abuse, learning disabilities, personality disorders, and impulse control disorders. Furthermore, ADHD may have a serious impact on education, employment and social functioning [9-15].

Twin and adoption studies have shown that the mean heritability of ADHD accounts for about 75% of the variance in symptoms suggesting that genetic factors play an important role in the aetiology of ADHD [16]. However, identifying susceptibility genes for ADHD is still difficult, because ADHD is a complex and heterogeneous disorder not only with respect to clinical diagnosis and treatment but also in terms of genetic and environmental causes and their interactions [16,17]. As a consequence, large samples are needed in order to have sufficient power for the detection of genetic variants implicated in ADHD [18,19]. Collaboration between several research centres is a method for increasing the size of a study sample without increasing the time of data collection. The International Multicentre ADHD Genetics (IMAGE) project included 11 centres in 8 countries in the collection of behavioural data from 1400 European sibling pairs and genetic data on the children and their parents. Moreover, the IMAGE project provides a large database for future research because cell lines containing DNA from the sample have been stored http://www.nimhgenetics.org and allow infinite DNA replication for future genetic analyses [20].

Until now, a variety of different analyses based on the IMAGE dataset or parts of it including molecular genetic studies have been published. These studies investigated the genetic association or linkage to ADHD [21-32], comorbidities [33-38], intelligence [39], neuropsychology [40-42], season of birth [43], parent of origin effect [44], age of ADHD onset [22], parental expressed emotion [45], and genetic population differences [46]. A periodically updated list of IMAGE publications is available at the IMAGE homepage http://image.iop.kcl.ac.uk/. The present contribution presents a comprehensive description and analysis of the diagnostic profile of those children who completed the full diagnostic process, including the interview, i.e., all 1068 probands of the IMAGE sample and the 339 siblings who were suspected to have ADHD.

Data were collected from different centres to enlarge the sample size and. hence, gaining power in statistical analyses. However, the subsamples of the different centres may differ from each other in numerous aspects in spite of standardized recruiting procedures, leading to a greater heterogeneity and a loss of statistical power. Thus, in multi-centre studies like the IMAGE project one might arrive at a conflict between a gain in statistical power by enlarging the sample size and a loss of power due to greater variance of data stemming from differences between centres.

The diagnostic procedure may be another source of heterogeneity which is, more difficult to measure and control in comparison to the variance due to centre differences. The IMAGE project used DSM-IV diagnostic criteria which required probands to a pre-defined symptom threshold along with meeting criteria for age at onset and impairment [3]. Particularly in genetic analyses, it is important to account for possible discrepancies between the variation of ADHD symptoms with age and gender in the population, and a symptom based diagnostic procedure which is insensitive to these effects to a large extent. Consequently, children with an identical diagnostic profile but of different age or gender may differ systematically from each other not only with respect to their deviation from age and gender specific population means but also by their genetic profile.

The present analyses investigated the individual contribution of each DSM-IV ADHD symptom to the discrimination between probands and unaffected siblings. It also identified factors influencing the operational decision on the presence of a single symptom. Furthermore, there was a specific interest in the analyses of informant effects (parent vs. teacher ratings) and diagnostic instrument effects (interview vs. questionnaire) on frequencies of each of the 18 DSM-IVADHD symptoms. To summarize, findings based on the following analyses will be presented:

- differences in age and sample size across gender, family status, and centres

- differences in the number of symptoms and differences in the age the first symptom was detected across gender and diagnostic subtypes

- comparison of frequencies of diagnostic subtypes across centres in the sibling sample

- differences in medication across diagnostic subtypes and centres

- comparison of centre effects on mean symptom frequencies across all 18 DSM-IV ADHD symptoms

- informant effects on each of the 18 diagnostic ADHD symptoms

- differences between interview and questionnaire ratings on each of the 18 DSM-IV ADHD symptoms

- discriminant diagnostic strength of all ADHD symptoms

- centre and gender effects on comorbid symptoms in probands.

A comprehensive analysis of the dimensional behavioural measures of the IMAGE sample, i.e. the questionnaire scores and the IQ findings in the whole IMAGE sample of 1068 probands and 1446 unselected siblings, is provided in a companion paper [47].

## Methods

### Participants and study protocol

The participating families were recruited between April 2003 and April 2007 in 11 European specialist ADHD centres: Amsterdam (NLD_A), Dublin (IRL_D), Essen (GER_E), Gent (BEL_G), Göttingen (GER_G), Jerusalem (ISR_J), London and Southamptom (ENG_L/S), Nijmegen (NLD_N), Petah Tiqva (ISR_P), Valencia (ESP_V), and Zürich (SWI_Z). Approval was obtained by the Institutional Review Board of SUNY Upstate Medical University and from ethical review boards within each country. Informed consent was obtained for the use of the samples for analyses related to the genetic investigation of ADHD. Recruited families had at least one child with diagnosed or suspected combined type Attention Deficit-Hyperactivity Disorder (ADHD-CT) as defined in the DSM-IV manual [3]. This restriction on the combined subtype was chosen due to the genetic focus of the IMAGE project [19]. Further entry criteria for assessment were: white Caucasian ethnicity of all participants, availability of one or more sibling, children between the ages of 5 and 17 years, participation of a minimum of four family members including one parent, and consent of all persons to give blood samples or buccal swabs for DNA extraction.

Families were excluded from genetic analyses, if either the proband or the participating sibling had an IQ<70, a diagnosis of schizophrenia or autism, a neurological disorder of the central nervous system or a genetic disorder that might mimic ADHD based on both history and clinical assessment. Children with classical or atypical autism were excluded from the IMAGE project because some genetic regions are known to be associated both with autism and ADHD [19]. There was no rule for assigning proband status to a certain child of a family when several children fulfilled criteria for ADHD-CT. In general, the researchers defined the child with the highest probability to fulfil the criteria to be the proband, and only swapped the roles if the designated proband did not meet the criteria and, at the same time, a designated sibling met the criteria.

In the study design of the IMAGE project, the genetic analyses were based on the comparison between ADHD probands and their 'unselected' siblings. In fact, the sibling group in the genetic analyses should contain children with ADHD symptoms of the whole continuum, except those with an ADHD-Diagnosis [19]. The full diagnostic procedure, particularly the interview, was, therefore, applied to the siblings only in case of suspected ADHD, i.e., (a) if they had a clinical diagnosis of ADHD, (b) if the recruiting clinician suspected ADHD, (c) if the sibling achieved a T-score of >63 in either the parents' or the teachers' N-subscale ('DSM-IV: total') of the Conners' questionnaire, or (d) if the sibling was taking stimulant medication. In contrast to the proband group, only the ADHD part of the interview was used for siblings.

In the present publication and in its companion paper [47] all 1446 siblings remained in the sample, regardless of their ADHD diagnosis. However, due to the described conditions of diagnosis in siblings, all analyses based on diagnostic data were restricted to the probands and the 339 siblings, who underwent the diagnostic procedure.

### Measures

#### Diagnostic Interview

To assess children's symptoms more objectively than by questionnaires, Taylor and associates developed a standardized, semi-structured interview, the Parental Account of Childhood Symptom (PACS), which was used in a slightly adapted version in this study [48-50].

At least one interviewer per participating centre underwent comprehensive training by a team under the supervision of Eric Taylor at the London Institute of Psychiatry (IoP), including cross validation of videotapes and interviews with parents of ADHD children referred to the IoP. If additional interviewers were used, each centre was responsible for their training and supervision. The interviewers were child psychiatrists or clinical child psychologists. The average inter-rater agreement across all centres was 96.6%, and the mean kappa coefficient was 0.88 (range 0.71-1.00) [29].

In the PACS interview parents are asked to rate the behaviour of their child not in terms of deviance from normality, but rather by describing the behaviour according to its frequency ('How often does the child usually leave the seat during mealtimes?') or severity ('What does the child do when in a temper?'). The interviewer then matches the parent's statement to a scale with specific categories for each question. The frequency categories of e.g., 'restlessness at mealtimes' are: (0) no restlessness, (1) leaving seat once only, (3) leaving seat 2 to 5 times (4) leaving seat > 5 times. The severity categories of e.g., 'severity of loss of temper', are the following: (0) no loss of temper, (1) mild: shouts, waves arms, stamps feet, (2) marked: throws things, kicks objects, (3) severe: breaks things, kicks or hits people. The PACS consists of four sections: (1) emotional patterns, (2) activity level and inattentive behaviour, (3) disruptive behaviour, and (4) comorbid and other problems.

The ADHD-section of the PACS, which was used to confirm the ADHD combined type diagnosis, covers ADHD related behaviour in different situations (watching TV, reading, playing alone, playing with friends, mealtimes, shopping, family outings, home-task, home-work). Depending on the situation, the parents have to rate the frequency or severity of their child's hyperactivity-related behaviour (leaving seat, fidgeting, talking, making noise etc.), inattention-related behaviour (attention to details, making mistakes, listening to instructions, following instructions, being distracted, organising, etc.), and impulsive behaviour (impatience while waiting, interrupting, etc.). A specific algorithm combines and weighs the rated behaviour across situations finally leading to a dichotomous statement about the presence or absence of the corresponding DSM-IV symptom. To check for other diagnostic criteria, such as, questions about age of symptom detection, parental perception of syndrome severity, clinically significant impairment, and problems at school are asked afterwards with respect to both the inattentive section and the hyperactivity/impulsivity section. Each major section ends with questions about the parental coping with the children's problems.

Whenever possible, the ADHD section of the PACS focused on behaviour when the child was not medicated. To control the influence of medication on the ADHD section of the PACS, the medication status associated with the rated behaviour was recorded in a variable with five levels: (1) current behaviour, not under medication, (2) behaviour during a one-week-period off medication, (3) behaviour during intermittent days off medication, (4) retrospective assessment of behaviour due to constant use of medication, and (5) behaviour while medicated. For further analyses a secondary dichotomous variable (MED2), with the levels 'medicated behaviour' and 'unmedicated behaviour', was generated by collapsing the variable levels (1) to (4) of the primary medication status variable into one category.

The sections dealing with emotional problems (depression, anxiety) and disruptive behaviour (oppositional defiant disorder and conduct disorder) in the PACS are structured similarly to the ADHD-section except that symptoms are not evaluated across multiple situations. The fourth section assesses co-morbid disorders (Tourette's Syndrome, bipolar affective disorder, substance misuse disorders, obsessive compulsive disorder, attachment disorders, schizophrenia, and 'other psychiatric disorders) at a syndrome level except autism spectrum disorders, which are assessed at a symptom level. Finally, the positive and negative expressed emotions of the interviewed parents are rated by the interviewer.

#### Questionnaires

The Conners' ratings scales for parents and teachers (CPRS-R:L, CTRS-R:L) [51], the Strength and Difficulties Questionnaires (SDQ, parent and teacher version) [52], and the Social Communication Questionnaire (SCQ, parent version) [53] were assessed in all participating children. Each of the two Conners' questionnaires (CPRS-R:L and CTRS-R:L) contains a subset of 18 questions covering the DSM-IV ADHD symptoms. This subset was used as a symptom checklist in the diagnostic procedure (see section on abbreviations for a detailed description of the symptoms and the section on the diagnostic procedure for the detailed diagnostic algorithm). The N-subscales ('DSM-IV: total') of both the CPRS-R:L and CTRS-R:L were used as a screening instrument for applying the ADHD diagnostic procedure in the siblings. Similarly, the SCQ was used as a screening instrument for applying the autism section of the PACS in probands and siblings.

The dimensional measures of all Conners' scales, the scales of the SDQ and the SCQ, and the IQ measures are described and analysed in the companion paper [47].

#### Intelligence assessment

Intelligence (IQ) measures were either assessed separately, or in combination with further neuropsychological testing, depending on the participation of each study centre in the neuropsychological part of the study [41]. Former IQ test results were used instead, if the tests were not older than one year. Children had to be off stimulant medication for 48 hours before IQ testing.

#### Diagnostic procedure and criteria

All parent and teacher questionnaires were used in the complete sample. The probands' behaviour at home was additionally assessed by the full PACS interview with their parents, except for the autism section of the interview that was administered to probands and siblings only if their SCQ score was 14 or higher. In contrast to the probands, only the ADHD section of the PACS was applied in those siblings who were suspected to have ADHD according to the criteria described above.

The DSM-IV diagnosis of ADHD was based on the CTRS-R:L and the PACS interview. A DSM-IV symptom list was generated by combining the DSM-IV symptoms from the PACS with the 18 DSM-IV items of the CTRS-R:L. A symptom was rated as present if either the diagnostic criterion of the specific PACS algorithm combining and weighing the responses to the symptom-related questions was met, or if the corresponding DSM-IV item of the CTRS-R:L was coded 2 or 3. To diagnose ADHD-CT among probands, DSM-IV criteria for both the inattention subtype and the hyperactivity/impulsivity subtype had to be met, i.e., 6 out of 9 inattention symptoms (abbreviated IA1 to IA9), 6 out of 6 hyperactivity symptoms (abbreviated HYP1 to HYP6) and 3 impulsivity symptoms (abbreviated IMP7 to IMP9) (see the abbreviations section for a detailed description of the symptoms and the abbreviations used hereafter). Additional diagnostic DSM-IV criteria including age of symptom onset below the age of 7 years, or absence of other psychiatric or neurologic disorders which may cause ADHD symptoms, were derived from the PACS interview. Pervasiveness was fulfilled if at least 2 symptoms of both the PACS and the CTRS-R:L were present, or if symptoms were rated as present in 2 or more different situations of the PACS interview. Clinical impairment was inferred by the fact that at least 12 symptoms exceeded the diagnostic threshold, and additionally was verified in the PACS interview.

The diagnoses of classical or atypical autism leading to the exclusion of a child from the study were defined by a specific algorithm based on the interview data of the PACS autism section.

### Statistical procedures

Most of the continuous variables examined were skewed and the various subsamples had unequal variances and unequal sample sizes. In particular, the questionnaire data were not only heavily skewed, but also skewed in opposite directions in probands and siblings. The assumptions of normality and homoscedasticity, which should be met for parametric statistical analysis, were violated for almost all continuous variables. Simulations have shown that even small deviations from normality can cause strong differences between the actual and the nominal Type I error and can result in low power, even with large sample sizes [54-58]. Therefore, the present investigation applied statistics that are robust to deviations from normality, symmetry, and heteroscedasticity.

- The percentile bootstrap procedure *trimpb *[58,59], with 2000 bootstrap samples, was applied to compute robust confidence intervals (CI's) for means and trimmed means in R [60].

- Chi-square-tests [61] were used for the analysis of two-dimensional contingency tables.

- Hierarchical log-linear analyses with backward elimination [61] were used for multidimensional contingency tables. As lower order effects in hierarchical models always are confounded with higher order interactions, only effects of the highest order will be reported.

- Robust two-way and three-way analyses were calculated in R by applying the procedures *t2way *and *t3way *[57,59] methods for trimmed means with estimates of standard errors and degrees of freedom adjusted for trimming, unequal variances and unequal sample sizes. This method provides a test value ('Q') which can be used to test null-hypotheses of main effects and interactions, and adjusted critical values ('crit.') for the 1-alpha quantile of a chi-square distribution. When these analyses are based on residuals of the dependent variable on age, the test value is named 'QRES'.

- Robust post-hoc pairwise comparisons were computed in R by using the bootstrap procedure *linconb6 *[62], an expansion of the procedure *lincon *[57], which allows unequal variances; 599 bootstrap samples were taken by default; CI's with family-wise 95% coverage probability level were calculated to control the false positive error rates associated with performing multiple statistical tests.

- Binary logistic regression analyses [61] were computed when information was measured in terms of frequencies. This procedure was applied to identify the contribution of independent variables to group differences.

- Residuals of a linear regression of target variables on age were calculated [61] for use in further statistical procedures in order to adjust the results for age effects.

## Results

### Sample characteristics

#### Sample size

After applying all inclusion and exclusion criteria, the sample consisted of 1068 probands and 1446 siblings, significantly differing in size from each other χ^2^=57.1, df = 1, p < .001 (Table 1). Boys and girls were equally distributed among the siblings (730 boys, 716 girls), but not among the probands (938 boys, 130 girls), resulting in a significant gender effect on sample size, χ^2^=268.8, df = 1, p < .001, and a significant gender by proband status interaction effect on sample size, χ^2^=387.7, df = 1, p < .001. The sample sizes of the 11 centres ranged from 81 to 431 and significantly differed across centres, χ^2^=758.2, df = 10, p < .001. No higher order interaction effect on sample size including the centre variable was significant, indicating equal gender ratios and equal proband/sibling ratios across centres.

**Table 1:** Sample size * and Age°, divided by family status, gender, and centre

		***Probands***				***Siblings***				***All***			
				
		***Male***	***Female***	***All***	***Sig ^§^***	***Male***	***Female***	***All***	***Sig ^§^***	***Male***	***Female***	***All***	***Sig ^§^***
				
BEL_G	N	27	5	32		36	13	49		63	18	81	
	Age(m)	10.7	12.2	10.9		11	9.1	10.5		10.9	9.9	10.7	
	Age(SD)	2.8	1.6	2.7		3.3	3.1	3.3		3.1	3.1	3.1	
													
ENG_L/S	N	164	15	179		122	130	252		286	145	431	
	Age(m)	11.6	12.7	11.6	^1+^	10.7	11	10.9		11.2	11.2	11	^1+^
	Age(SD)	2.8	2.4	2.7		3.3	3.3	3.3		3	3.2	3.1	
													
ESP_V	N	69	5	74		40	35	75		109	40	149	
	Age(m)	9.4	8.6	9.4	^1-, 2-^	11.1	11.9	11.5		10	11.5	10.4	
	Age(SD)	2.4	3.3	2.4		2.8	3	2.9		2.6	3.2	2.9	
													
GER_E	N	32	4	36		23	26	49		55	30	85	
	Age(m)	10.8	10.5	10.7		10.1	11.8	11		10.5	11.6	10.9	
	Age(SD)	2.9	2.6	2.8		3.8	4.2	4		3.2	4	3.5	
													
GER_G	N	76	6	82		54	56	110		130	62	192	
	Age(m)	10.4	9.7	10.3	^1-^	10.2	10.3	10.2		10.3	10.2	10.3	
	Age(SD)	2.3	2.3	2.3		3.2	3.7	3.5		2.7	3.6	3	
													
IRL_D	N	85	15	100		70	73	143		155	88	243	
	Age(m)	11.4	10.5	11.2	^2+^	10.1	11	10.6		10.8	10.9	10.8	
	Age(SD)	3.2	2.9	3.2		3.1	3.1	3.1		3.2	3.1	3.2	
													
ISR_J	N	52	8	60		27	40	67		79	48	127	
	Age(m)	10	8.3	9.8	^1-, 3-^	10.9	10.3	10.5		10.3	9.9	10.2	
	Age(SD)	2.7	1.2	2.6		3.4	3.3	3.3		2.9	3.2	3	
													
ISR_P	N	120	13	133		109	87	196		229	100	329	
	Age(m)	10.4	11	10.4	^1-^	11.6	11.4	11.5		11	11.4	11.1	^1+^
	Age(SD)	2.8	3.7	2.9		3.4	3.3	3.4		3.1	3.4	3.2	
													
NLD_A	N	135	20	155		106	109	215		241	129	370	
	Age(m)	11.2	10.7	11.1	^2+, 3+^	10.4	11.1	10.8		10.8	11	10.9	
	Age(SD)	2.7	2	2.6		3.4	3.9	3.6		3	3.6	3.2	
													
NLD_G	N	135	30	165		116	114	230		251	144	395	
	Age(m)	11.1	10.4	11	^2+,3+^	10.9	10.5	10.7		11	10.5	10.8	^1+^
	Age(SD)	2.7	3.5	2.8		3.2	3.5	3.3		2.9	3.5	3.1	
													
SWI_Z	N	43	9	52		27	33	60		70	42	112	
	Age(m)	9.8	9.8	9.8	^1-^	10	9.3	9.6		9.9	9.4	9.7	^1-^
	Age(SD)	1.7	2.3	1.8		3.9	2.7	3.2		2.7	2.6	2.6	
													
All	N	938	130	1068		730	716	1446		1668	846	2514	
	Age(m)	10.8	10.6	10.8		10.7	10.8	10.8		10.8	10.8	10.8	
	Age(SD)	2.7	2.9	2.8		3.3	3.5	3.4		3	3.4	3.1	

* Significant main effects of status, gender, and centre, and interaction effect of status × gender (see text)

° Significant main effect of centre, and interaction effect of status × centre (see text)

§ Significant age differences within each column between pairs with equal number and different sign (e.g. 3+ and 3-)

#### Age

he mean age of the total sample was 10.8 years (SD = 3.1years). A three way analysis of variance including gender, family status, and centre revealed no main effects of gender and status on age, but a significant main effect by centre, Q = 44.9, crit.=20.8, p < .001. Post hoc pairwise comparisons between centres with a 5% family-wise error rate revealed that the children of SWI_Z were significantly younger than those of three other centres, namely NLD_G (CI = 0.01-2.22 years), ISR_P (CI = 0.16-2.48 years), and ENG_L/S (CI = 0.50-2.67 years). There was a significant centre by status interaction effect on age, Q = 34.8, crit.=20.7, p < .001. On the one hand, none of the 55 post hoc pairwise comparisons in the sibling sample were significant (probability level adjusted for multiple tests). On the other hand, ten pairwise comparisons between centres within the proband sample differed significantly as indicated by non-overlapping 95% family-wise CI's between centres (Figure S1 in Additional file 1). No other interaction effects including centre, gender, or status on age were significant. This indicates that age differences between boys and girls (whether significant or not) were not dependent on status or centre.

### ADHD subtypes, symptom quantity, and age at symptom detection

#### Symptom load in probands

The mean number of inattentive symptoms (20% trimmed mean), based on the PACS interview and the Conners' teacher questionnaire, was 8.5 in boys and 8.3 in girls, and the mean number of hyperactive/impulsive symptoms was 8.5 in boys and 8.4 in girls (see Table 2). Robust two-way analyses of centre and gender effects on the (20% trimmed) mean number of ADHD symptoms were conducted. There were significant gender effects on the number of inattentive (Q = 4.85, p = .03), but not of hyperactive/impulsive symptoms. In addition, there were highly significant centre effects on inattentive symptoms (Q = 88.37, p < .001), hyperactive/impulsive symptoms (Q = 93.53, p < .001), and a significant gender by centre effect for the number of inattention symptoms (Q = 103.8, p < .001) but not for the number of hyperactive/impulsive symptoms.

**Table 2:** ADHD subtypes, symptom frequencies and age of symptom onset

			Number of symptoms *								**Age at first symptom detection°**							
**Boys**																		
			***Inattention***				***Hyperactivity/Impulsivity***				***Inattention***				***Hyperactivity/Impulsivity***			
***Status***	***ADHD subtype***	***N***	***Mean _t_***	***CI low***	***CI _up_***	***Range***	***Mean _t_***	***CI low***	***CI _up_***	***Range***	***Mean _t_***	***CI low***	***CI _up_***	***Range***	***Mean _t_***	***CI low***	***CI _up_***	***Range***
				
Siblings	No Diagnosis	39	6.28	5.24	7.24	0 - 9	4.60	3.32	5.92	1 - 9	6.20	5.00	7.75	1 - 15	3.50	2.13	5.19	1 - 13
	Hyperactive/Impuslive	15	4.33	3.56	5.00	1 - 5	7.89	6.89	8.56	6 - 9	4.75	3.63	5.75	1 - 9	2.89	2.11	3.78	1 - 6
	Inattentive	43	7.59	7.11	8.07	6 - 9	3.96	3.41	4.41	1 - 5	4.38	3.85	4.96	1 - 6	3.67	2.90	4.43	0 - 10
	Combined	118	8.47	8.21	8.69	6 - 9	8.40	8.17	8.56	6 - 9	4.06	3.63	4.44	1 - 10	2.94	2.49	3.39	1 - 7
	All subtypes	215	7.88	7.60	8.13	0 - 9	7.22	6.74	7.60	1 - 9	4.42	4.14	4.70	1 - 15	3.11	2.74	3.46	0 - 13
				
Probands	Combined	938	8.50	8.42	8.58	6 - 9	8.47	8.39	8.55	6 - 9	4.23	4.10	4.35	0 - 12	2.41	2.27	2.55	0 - 11
				
																		
**Girls**																		
			***Inattention***				***Hyperactivity/Impulsivity***				***Inattention***				***Hyperactivity/Impulsivity***			
***Status***	***ADHD subtype***	***N***	***Mean _t_***	***CI low***	***CI _up_***	***Range***	***Mean _t_***	***CI low***	***CI _up_***	***Range***	***Mean _t_***	***CI low***	***CI _up_***	***Range***	***Mean _t_***	***CI low***	***CI _up_***	***Range***
				
Siblings	No Diagnosis	40	4.21	3.33	5.17	0 - 9	3.04	2.21	3.83	0 - 9	6.32	5.32	7.63	1 - 16	4.71	2.86	7.29	1 - 15
	Hyperactive/Impuslive	11	4.00	2.57	4.71	1 - 5	6.86	6.29	7.57	6 - 9	3.60	2.40	6.80	1 - 11	3.57	2.14	5.00	1 - 6
	Inattentive	33	7.38	6.95	7.86	6 - 9	3.24	2.43	3.90	1 - 5	5.26	4.74	5.74	1 - 6	3.40	2.20	4.60	1 - 6
	Combined	40	8.33	7.92	8.71	6 - 9	8.38	7.96	8.71	6 - 9	4.13	3.29	4.83	0 - 8	2.42	1.75	3.29	0 - 6
	All subtypes	124	6.76	6.22	7.30	0 - 9	5.20	4.50	5.88	0 - 9	4.91	4.49	5.31	0 - 16	3.16	2.53	3.81	0 - 15
				
Probands	Combined	130	8.27	8.04	8.49	6 - 9	8.38	8.13	8.62	6 - 9	4.10	3.75	4.43	0 - 12	1.97	1.64	2.35	0 - 11
				
																		
**All**																		
			***Inattention***				***Hyperactivity/Impulsivity***				***Inattention***				***Hyperactivity/Impulsivity***			
***Status***	***ADHD subtype***	***N***	***Mean _t_***	***CI low***	***CI _up_***	***Range***	***Mean _t_***	***CI low***	***CI _up_***	***Range***	***Mean _t_***	***CI low***	***CI _up_***	***Range***	***Mean _t_***	***CI low***	***CI _up_***	***Range***
				
Siblings	No Diagnosis	79	5.27	4.49	5.98	0 - 9	3.63	2.96	4.35	0 - 9	6.19	5.35	7.24	1 - 16	3.93	2.87	5.43	1 - 15
	Hyperactive/Impuslive	26	4.19	3.50	4.69	1 - 5	7.38	6.81	8.06	6 - 9	4.31	3.38	5.46	1 - 11	3.13	2.38	3.94	1 - 6
	Inattentive	76	7.50	7.13	7.89	6 - 9	3.67	3.17	4.11	1 - 5	4.80	4.36	5.20	1 - 6	3.56	2.85	4.24	0 - 10
	Combined	158	8.44	8.23	8.66	6 - 9	8.40	8.19	8.55	6 - 9	4.07	3.73	4.44	0 - 10	2.81	2.41	3.22	0 - 7
	All subtypes	339	7.56	7.27	7.79	0 - 9	6.53	6.10	6.92	0 - 9	4.59	4.36	4.82	0 - 16	3.09	2.78	3.41	0 - 15
				
Probands	Combined	1068	8.48	8.39	8.55	6 - 9	8.46	8.38	8.54	6 - 9	4.22	4.10	4.33	0 - 12	2.36	2.23	2.48	0 - 11

* Frequencies are based on the combination of the parental Interview (PACS) and the teacher questionnaire (CTRS)

° As reported by the PACS

*Mean _t _*20% trimmed mean

*CI low *95% confidence interval for trimmed mean (lower end)

*CI _up _*95% confidence interval for trimmed mean (upper end)

Because age correlated negatively with the number of hyperactive symptoms (Spearman's rho = -.124, p < .001), a similar analysis was calculated based on age-adjusted number of hyperactive/impulsive symptoms (residuals). Similar to the analysis of unadjusted number of symptoms, this analysis revealed significant centre effects only on number of hyperactive symptoms (Q_RES _= 65.29, p < .001).

Post hoc analyses of the number of symptoms between centres showed that the mean number of symptoms was lowest in the SWI_Z subsample both for inattention (7.9) and hyperactivity-/impulsivity (7.5), and highest in the GER_G subsample both for inattention (8.9) and hyperactivity (8.9). Pairwise comparisons of number of symptoms between centre sub-samples revealed six centre pairs differing significantly from each other in the inattention domain and five in the hyperactive/impulsive domain (probability level adjusted for multiple tests). The graphs in Figure S2 in the Additional file 2 show the mean symptom numbers at each centre, all significant pairwise differences (probability level adjusted for multiple tests), and the gender by centre interactions. Post hoc analyses of age-adjusted centre effects on the number of hyperactive symptoms revealed minor changes in rank order of centres with medium symptom numbers (ESP_V, ISR_J, NLD_A, GER_E). All significant paired differences between centres remained significant, and, additionally, the centre GER_G had significantly more symptoms than the centres ISR_J, IRL_D, and BEL_G. This finding indicates that the hyperactive/impulsive symptom numbers differed to a greater extent between centres, when age effects were removed from the analysis.

#### Age at symptom detection in probands

The mean age at inattention symptom detection was 4.2 years in boys and 4.1 years in girls. Similarly, girls were younger (2.0 years) at first detection of hyperactive/impulsive symptoms than boys (2.4 years).

No significant gender effects were found in a two-way analysis of centre and gender on the age at symptom detection. The first inattentive symptom occurrence differed between centres (Q = 93.73, p < .001) as well as the first hyperactive/impulsive symptom occurrence (Q = 58.08, p < .001). A centre by gender interaction significantly influenced the age at first detection of inattentive symptoms (Q = 32.1, p = .017), but not of hyperactive/impulsive symptoms.

Because the age of the probands significantly correlated with the age of first inattentive symptom occurrence (Spearman's rho = .132, p < .001), a similar analysis was performed on age-adjusted detection of inattentive symptoms (residuals). The results of this age adjusted analysis were similar to the non-adjusted analysis: the centre effect (Q_RES _= 82.66, p < .001) and the centre by gender interaction effect (Q_RES _= 28.73, p = .028) was significant, indicating that the parents' estimates of the first inattention symptoms differed between centres, independent of the actual age of the probands, and that gender effects varied across centres.

Post hoc analyses of centre differences regarding inattention symptom detection (Figure S2 in Additional file 2) showed that the occurrence of inattention symptoms was perceived earliest by parents of the NLD_A sample (3.4 years) and latest by those of the ISR_P sample (5.3 years). Hyperactive/inattentive symptoms were perceived earliest by the parents of the NLD_G sample (1.6 years), and latest by those of the ISR_P sample (4.0). Out of 55 post hoc analyses of inattention symptom detection, there were twelve significant differences between centres (probability level adjusted for multiple tests). In the hyperactivity/impulsivity domain there were nine centre pairs differing significantly from each other. Figure S2 in Additional file 2 shows the mean symptom detection ages for all centres and all significant pair differences. In addition, the significant gender by centre interaction for inattention symptom detection is illustrated graphically.

Age-adjusted post hoc analyses of centre effects on age of inattention detection revealed small changes compared to the analyses based on raw scores: two pairs of adjacent centres according to rank (BEL_G and ENG_L/S, and ESP_V and IRL_D) changed their rank position, and the centre IRL_D no longer differed significantly from centres ISR_J and NLD_G (see Figure S2 in Additional file 2).

#### ADHD subtypes in siblings

Interview data were available for 215 male and 124 female siblings. The diagnostic procedure resulted in 158 (47%) of these 339 siblings having combined type (ADHD-CT), 76 (22%) having inattentive type (ADHD-IT), 26 (8%) having hyperactive/impulsive type (ADHD-HT), and 79 (23%) having no ADHD diagnosis (ADHD-ND). The latter subtype resulted from number of symptoms below the diagnostic threshold (see Table 2). The percentage of boys was 75% among the 158 siblings with ADHD-CT, 58% among 26 siblings with ADHD-HT, 57% among 76 siblings with ADHD-IT, and 49% among the 79 siblings without diagnosis.

There were notable differences in subtype frequencies across centres. For instance, there was one subsample (ESP_V) consisting of siblings with ADHD-CT only, two sub-samples (BEL_G and ISR_J) containing no siblings with ADHD-HT, and one sample (GER_G) having no siblings with ADHD-IT (Table S1 and Figure S3 in Additional files 3 and 4).

A hierarchical loglinear analysis of gender and centre effects on the subtype frequencies in the sibling sample resulted in a model that retained main effects and two-way interactions, but no three-way interactions. The likelihood ratio of a goodness-of-fit test, χ^2^=27.32, df = 30, p = .607, indicated no significant difference between the predictions of the model and the data. Both two-way effects including the variable subtype, i.e. gender by subtype, χ^2^=89.25, df = 3, p < .001, and centre by subtype, χ^2^=88.38, df = 30, p < .001, were significant. Thus, the subtype frequencies differed between genders and across centres (see Figure S3 in Additional file 4), but the gender effects on subtype frequencies did not differ across centres.

#### Symptom load in diagnosed siblings (N = 339)

The mean number of inattentive symptoms (20% trimmed mean), based on the PACS interview and the Conners' teacher questionnaire, was highest in the CT subsample (8.4), followed by IT (7.5), ND (5.3), and HT (4.2) subsamples. Symptoms of hyperactivity/impulsivity were most frequent in CT (8.4), followed by HT (7.4), IT (3.7), and ND (3.6). Table 2 shows means and 95% confidence intervals for the population trimmed means, divided by family status and gender, and across diagnostic subtypes.

A two-way ANOVA revealed significant gender effects and subtype effects on symptom numbers for both inattentive and hyperactive/impulsive symptoms, but no gender by subtype interaction effects. Inattentive symptoms were more frequent in male siblings compared to female siblings (Q = 6.77, p = .012) and differed between subtypes (Q = 206.6, p < .001). Similarly, male siblings had more hyperactive/impulsive symptoms than female siblings (Q = 7.61, p = .008), and the symptom numbers differed between subtypes (Q = 353.6, p < .001; see Table 2). Because the siblings' age correlated negatively with the number of hyperactive symptoms (Spearman's rho = -.275, p < .001), the effects of gender and subtype on age adjusted hyperactive symptom numbers (residuals) were additionally calculated. Similar to the non-adjusted analyses, this analysis revealed significant gender effects (Q_RES _= 11.20, p = .002) and subtype effects (Q_RES _= 438.9, p < .001) on the number of symptoms present, with an additional gender by subtype interaction effect (Q_RES _= 8.89, p = .045).

#### Age at symptom detection in siblings

The parents mean retrospective estimate of the siblings' age (20% trimmed mean) when symptoms were present for the first time was lowest in siblings with ADHD-CT (inattention: 4.1 years, hyperactivity/impulsivity: 2.8 years) and highest in children without an ADHD diagnosis (inattention: 6.2 years, hyperactivity/impulsivity: 3.9 years; Table 2).

In two-way analyses, the first occurrence of ADHD-DSM-IV symptoms in these 339 siblings did not differ between boys and girls, neither for inattentive nor for hyperactive/impulsive symptoms. A subtype effect on the age of symptom detection was present with regard to inattentive symptoms (Q = 18.9, p = .002) but not with regard to hyperactive symptoms; gender by subtype interaction effects on age at detection were not significant in both symptom groups of the sibling sample. Because the reported age of inattention symptom detection correlated with the age of the siblings (Spearman's rho = .211, p < .001), the same analysis was calculated based on age adjusted first occurrence of inattentive symptoms. Similar to the original analysis, only the main effect of subtype was significant (Q_RES _= 13.8, p = .009).

### Medication status in the PACS interview

For 1400 children, information about stimulant medication during the period rated by their parents was available: 434 (31.0%; boys: girls ratio = 2.4:1) were permanently off medication, 186 (13.3%; 6.8:1) were off medication for one week, 446 (31.8%; 7.0:1) were off medication on intermittent days, 202 (14.4%; 6.8:1) were rated retrospectively due to constant use of medication, and 132 (9.4%; 7.8:1) were rated during a period of permanent medication. In summary, the interview data of 1268 (90.6%; 4.4:1) children was based on unmedicated periods, while it was based on medicated periods for 132 (9.4%; 7.8:1) children.

The percentage of children rated under medication differed between ADHD subtypes. It was highest (10%) for ADHD-CT, followed by ADHD-ND (8.2%), ADHD-IT (3.9%), and ADHD-HT (0%). A total of 10.2% of the 1151 boys, but only 6% of the 249 girls were rated under medication. The percentage of children rated under medication also differed between centres. There were four centres with low percentages, namely ESP_V (0%), ISR_J (1.5%), ISR-P (2.1%), NLD_A (1.7); three centres with medium percentages, namely BEL_G (4.1%), GER_G (5.0%), IRL_D (3.0%); two centres with high percentages, namely GER_E (7.3%) and SWI_Z (7.6%), and two centres with percentages clearly above average, namely ENG_L/S (12.7%) and NLD_G (35%).

A statistical analysis using a hierarchical loglinear model including the dichotomized medications status (MED2; see methods section), the diagnostic subtype, family status, and gender resulted in a final model that retained only main effects and two-way interactions, but no three- and four-way interactions. The likelihood ratio for this model (χ^2^=8.374, df = 15, p = .908) did not indicate a significant difference between the model and the data. The gender by status interaction effect on MED2, (χ^2^=6.72, df = 1, p = .010) was the only significant effect when including the variable MED2 after the backward elimination procedure stopped. To break down this effect, chi-square tests on status and MED2 variables were performed separately for boys and girls. For boys, there was no significant association between family status and medication status, χ^2 ^= .027, df = 1, p = .87, whereas for girls this association was significant, χ^2^=7.6, df = 1, p = .006. In terms of odds ratios, male probands were 1.04 times more likely to be medicated than male siblings, whereas female probands were 6.5 times more likely to be medicated than female siblings.

When centre was entered into the model (likelihood ratio: χ^2^=92.61, df = 275, p = 1.000), only main effects and two-way interactions remained significant. The only significant two-way effect on MED2 after backward elimination was again for gender by status (χ_p_^2^=5.86, df = 1, p = .015) indicating that medication did not vary across centres. Thus, the significant centre by medication effect can be ignored because it was confounded with the significant higher order effect of gender by status by MED2 due to the hierarchical character of the model.

### Diagnostic symptoms in the probands' sample

#### Effects of informant and diagnostic instruments on diagnostic symptom frequencies

**Figure 1:**
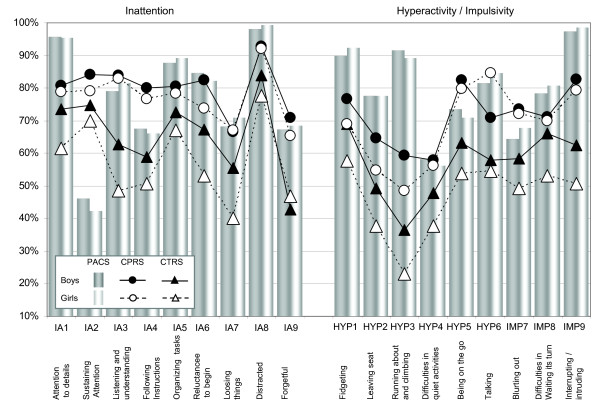
**Frequencies of ADHD symptoms in probands, assessed by parents (PACS, CPRS) and teachers (CTRS)**. Notes: All 1068 probands had an ADHD-CT diagnosis. Abbreviations: PACS: Parental Account of Clinical Symptoms, CPRS: Conners' Parent Rating Scales, CTRS: Conners' Teacher Rating Scales.

Frequencies of diagnostic symptoms in the proband sample, divided by gender and source of information are displayed in Figure 1. Beside the two sources used for the diagnostic procedure, namely, the parental interview (PACS) and the teacher questionnaire (CTRS), there were also symptom ratings available from the parents' questionnaire (CPRS).

The parent interview ratings (PACS) and the teacher questionnaire ratings (CTRS) contributed to the ADHD diagnosis. In boys and girls, all symptom frequencies were higher in the PACS compared to the CTRS, except for symptom IA2 (29% lower in boys and 28% lower in girls; see Figure 1). The PACS ratings for inattentive symptoms in boys were on average 11% higher than the CTRS ratings (15% higher, when IA2 was excluded). Ratings for inattentive symptoms in girls were on average 20% higher in the PACS compared to the CTRS (23% higher, when IA2 was excluded). Hyperactive/impulsive symptoms in boys were rated 22% higher in the PACS compared to the CTRS; in girls these ratings were rated 33% higher on average. All 18 symptoms had, on average, 17% higher frequencies in the PACS than in the CTRS in boys and 27% higher frequencies in girls. These numbers were 20% and 30%, respectively, when IA2 was excluded.

To calculate the 'pure' informant effect, teacher (CTRS) and parent (CPRS) questionnaires were compared. All symptoms were more frequent when rated by parents than by teachers, both in boys and girls. The mean difference across inattentive symptoms was 14% in boys and 20% in girls; across hyperactive/impulsive symptoms the differences were 14% in boys and 22% in girls.

A comparison of the two parental sources, i.e. PACS and CPRS, reflecting the effect of the diagnostic instrument revealed smaller effects across all symptoms compared to the differences which included different informants. The symptoms were equal or more frequent when recorded in the interview in 12/18 symptoms in boys (differences from 0% to 32%) and in 13/18 symptoms in girls (0% to 41%). The remaining symptoms with higher frequencies in the CPRS compared to the PACS were IA2 (38% in Boys, 37% in girls), IA3 (5%, 1%), IA4 (13%, 11%), IA9 (4% only in boys), HYP5 (9%, 9%), and IMP6 (9%, 4%), Mean differences across all 18 symptoms were 3% in boys and 6% in girls, both indicating higher frequencies in the interview compared to the questionnaire. When the outlier symptom IA2 was excluded from the calculation, these differences were larger in boys (5%) and in girls (8%).

When all three sources were entered in a non-parametric analysis of variance, there was a significant effect of the source on symptom frequency in each of the symptoms in the whole sample (Friedman χ^2 ^between 37.5 and 688.4, df = 2, all p < .001), when calculated separately in girls (χ^2 ^between 11.5 and 106.5, df = 2, all p < .001), and in boys (χ^2 ^between 27.6 and 582.6, df = 2, all p < .001). Pair-wise statistical parent vs. teacher comparisons (Wilcoxon test), i.e. 18 comparisons PACS vs. CTRS and 18 comparisons CPRS vs CTRS, revealed highly significant results for all parents vs. teacher comparisons (all p < .001, except p = .002 for *IMP8 *in the CTRS vs. CPRS comparison). The pair-wise comparisons of instruments reflecting the parents view (PACS vs. CPRS) were not significant for three symptoms (*IA7*, *IA9*, and *HYP4*), significant in two comparisons (*IA3*, p = .006; *IA6*, p = .043), and highly significant (p < .001) in the remaining 13 comparisons.

To evaluate gender and centre effects on symptom frequencies simultaneously, hierarchical log-linear analyses were performed for each symptom and separately for all three sources, i.e., PACS, CPRS, and CTRS. These analyses revealed no significant gender effects for all symptoms, but there were significant centre effects for all symptoms except in three inattention symptoms (*IA5*, *IA8*, and *IA9*), as assessed by the PACS interview. Slightly different effects resulted in symptoms that were obtained from the parent questionnaire (CPRS). All centre effects were highly significant (p < .001), except for *IMP7 *(p = .023), but no gender effects were significant except for *HYP3 *(p = .047, more frequent in boys) and for *HYP6 *(p = .001, more frequent in girls).

In contrast to these two parent ratings, teacher assessed symptom frequencies (CTRS) were higher in boys than in girls for 11 of the 18 symptoms, i.e. all but *IA2, IA4, IA5, IA9, HYP4*, *HYP6*, and *IMP7*). All these significant differences reflected higher frequencies in boys. The centre effects, however, were all significant and were similar to the effects found for the parent ratings. (Detailed statistical results are not reported here, but available on request. For detailed statistics reporting differences in symptom frequencies between centres, see the next 25 section on combined symptom frequencies, which were relevant for the diagnostic procedure).

#### Symptom frequencies based on the combination of PACS and CTRS

The following analyses are based on the combined symptom presence (see methods section for the diagnostic algorithm). Because the diagnosis was based on the combination of the parental interview (PACS) and the teacher Conners' questionnaire (CTRS), i.e., a symptom was counted as present when it was present either in the PACS or the CTRS.

The frequencies of combined symptoms in the proband sample stratified by centres are presented in Table 3. The overall mean symptom frequency across all centres and all symptoms was 91.3%. Symptom frequencies across all centres ranged from 81.9% to 99.8% for inattentive symptoms, and from 78.5% to 99.3% for hyperactive/impulsive symptoms. The SWI_Z centre had the lowest total frequency across all symptoms (85.4%), across inattentive symptoms (87.4%) and across hyperactive/inattentive symptoms (83.3%). In contrast, the GER_G centre had the highest frequencies across all symptoms (96.5%), across inattentive symptoms (95.9%) and across hyperactive symptoms (97.0%). For five inattention symptoms and six hyperactive/impulsive symptoms there was a significant centre effect (not adjusted for multiple tests).

**Table 3:** Sorted ADHD symptom frequencies in probands, divided by centre

		**Probands** **(N = 1068)**														**Controls** **(N = 79)°**	**Logistic** **regression §**	
				
		***SWI_Z***	***BEL_G***	***IRL_D***	***NLD_G***	***ISR_P***	***GER_E***	***NLD_A***	***ESP_V***	***ISR_J***	***ENG_L/S***	***GER_G***	***All centres***	***χ_p_*^2^**	***p***	***All centres***	***T_w _^#^***	***p***
		
Inattention																		
IA8	Distracted	100.0	100.0	100.0	100.0	99.2	100.0	100.0	100.0	100.0	99.4	100.0	***99.8***	4.8	n.s.	87.3	0.000	.988
IA1	Attention to details	100.0	100.0	98.0	98.8	100.0	100.0	99.4	100.0	100.0	99.4	100.0	***99.4***	8.0	n.s.	69.6	3.809	.051
IA5	Organising tasks	92.3	100.0	93.0	97.0	97.7	100.0	97.4	100.0	98.3	96.6	98.8	***97.1***	17.0	n.s.	58.2	5.336	.021
IA6	Reluctance to begin	94.2	84.4	93.0	86.1	94.7	94.4	96.1	95.9	93.3	95.5	100.0	***93.6***	29.4	**	60.8	0.219	.640
IA3	Listening	94.2	96.9	89.0	81.2	99.2	97.2	96.1	98.6	100.0	86.0	98.8	***92.4***	74.3	***	49.4	6.718	.010
IA4	Following instructions	75.0	71.9	89.0	80.0	92.5	80.6	92.9	87.8	96.7	91.6	90.2	***88.0***	36.7	***	51.9	0.151	.698
IA2	Sustaining attention	78.8	90.6	89.0	91.5	79.7	83.3	90.3	78.4	88.3	86.6	100.0	***87.5***	41.5	***	44.3	0.746	.388
IA7	Losing things	71.2	71.9	82.0	77.6	78.2	88.9	85.8	90.5	81.7	88.8	89.0	***83.1***	24.5	**	45.6	0.603	.437
IA9	Forgetful	80.8	84.4	80.0	79.4	77.4	86.1	84.5	82.4	80.0	83.8	86.6	***81.9***	6.2	n.s.	46.8	0.172	.678
		
Mean (inattention)		87.4	88.9	90.3	87.9	91.0	92.3	93.6	92.6	93.1	92.0	95.9	91.4	26.9		57.1	1.973	
Hyperactivity Impuslivity																		
		
IMP9	Interrupting/intruding	96.2	100.0	100.0	100.0	98.5	100.0	100.0	100.0	98.3	99.4	98.8	***99.3***	13.3	n.s.	77.2	1.766	.184
HYP1	Fidgeting	98.1	88.5	96.0	98.8	96.2	100.0	99.4	98.6	98.3	98.9	100.0	***98.4***	12.0	n.s.	44.3	7.215	.007
HYP3	Running about/climbing	84.6	87.5	93.0	93.3	97.7	91.7	96.1	93.2	98.3	96.6	100.0	***94.9***	26.7	**	34.2	10.569	.001
HYP5	Being 'on the go'	80.8	78.1	85.0	97.0	91.7	94.4	95.5	91.9	95.0	96.6	98.8	***93.2***	41.2	***	32.9	7.956	.005
IMP8	Difficulties waiting turn	92.3	96.9	92.0	97.0	88.7	94.4	92.9	87.8	90.0	88.3	98.8	***92.2***	23.9	**	40.5	3.856	.050
HYP6	Talking	82.7	93.8	95.0	95.2	86.5	91.7	88.4	97.3	86.7	93.3	97.6	***91.9***	26.8	**	58.2	1.641	.200
HYP2	Leaving seat	80.8	78.1	90.0	89.1	84.2	83.3	89.7	86.5	98.3	90.5	98.8	***89.0***	31.4	**	36.7	2.798	.094
IMP7	Blurting out	75.0	87.5	85.0	82.4	90.2	83.3	78.7	87.8	76.7	82.1	86.6	***83.2***	14.5	n.s.	45.6	0.920	.338
HYP4	Quiet activities	59.6	68.8	73.0	80.0	73.7	80.6	69.7	79.7	81.7	89.4	93.9	***78.5***	50.6	***	27.8	1.129	.288
Mean (hyperactivity/impulsivity)		83.3	86.6	89.9	92.5	89.7	91.0	90.0	91.4	91.5	92.8	97.0	91.2	91.4		44.16	4.205	
				
Overall Mean		***85.4***	***87.7***	***90.1***	***90.2***	***90.4***	***91.7***	***91.8***	***92.0***	***92.3***	***92.4***	***96.5***	91.3	36.9		50.63	3.09	

Notes: For abbreviations of sites see methods section; sites in ascending order (overall mean); symptoms in descending order (All sites) within subtypes

χ_p_^2 ^Partial chi-square of hierarchical logliniear analysis

*** p < .001 ** p < .01 * p < .05 n.s. p ≥ .05

° Diagnosed siblings without ADHD diangosis (ADHD-NT)

^§ ^R^2 ^= .272 (Cox & Snell), .689 (Nagelkerke). Model X^2 ^(18) = 363.6, p < .001.

^# ^T_w _= Wald statistics (b/S.E._b_)^2^

In the comparable control group (ADHD-ND sibling group, N = 79), the overall mean symptom frequency was 50.6% across all symptoms, 57.1% across inattentive symptoms, and 44.2% across hyperactive/impulsive symptoms. The frequencies in the control sample did not differ between centres due to small sample size (Table S1 in Additional file 3). In summary, the symptom frequencies in the ADHD-ND group were smaller when compared to the proband group. However, the mean frequency difference between inattention and hyperactivity/impulsivity was clearly larger (12.9%) in the control sample compared to the proband sample (0.2%).

A binary logistic regression analysis in which the proband sample (N = 1068) was compared to the control sample (ADHD-ND; N = 79) showed that hyperactive/impulsive symptoms contributed more (mean Wald test, *T_w_*=4.25) to the discrimination between probands and unaffected siblings than inattentive symptoms (mean *T_w_*=1.97). Among the symptoms discriminating significantly, 'running about' (*HPY3*; *T_w_*=10.57) was the symptom that discriminated most strongly, followed by 'being on the go' (*HYP5*; *T_w_*=7.96), 'fidgeting' (*HYP1*; *T_w_*=7.22), 'listening' (*IA3*; *T_w_*=6.72), 'organizing tasks' (*IA5 *; *T_w_*=5.24), 'difficulties waiting turn' (*IMP8*; *T_w_*=3.86), and 'attention to details' (*IA1*; *T_w_*=3.81). See Table 3 for detailed results.

### Comorbid symptoms in probands

The most frequent comorbid disorders in probands were oppositional defiant disorder (ODD; 64%), conduct disorder (CD; 24%), anxiety disorders (ANX; 44%), and mood disorders (MOOD; 15%) (see Table 4).

**Table 4:** Comorbidities in the probands sample (divided by centre and gender)

	Sites°											
	
	*BEL_G*	*ENG_L/S*	*ESP_V*	*GER_E*	*GER_G*	*IRL_D*	*ISR_J*	*ISR_P*	*NLD_A*	*NLD_G*	*SWI_Z*	*Total*
	
Oppositional Defiant Disorder *												
*Boys*	18/9/0 66.7%	131/29/4 79.9%	37/32/0 53.6%	24/8/0 75.0%	57/19/0 75.0%	57/27/1 67.1%	37/15/0 71.2%	74/46/0 61.7%	83/52/0 61.5%	73/60/1 54.5%	19/19/5 44.2%	610/316/11 65.1%
*Girls*	5/0/0 100%	14/1/0 93.3%	3/2/0 60.0%	2/2/0 50.0%	4/2/0 66.7%	8/7/0 53.3%	6/1/1 75.0%	4/9/0 30.8%	6/14/0 30.0%	17/12/1 56.7%	6/3/0 66.7%	75/53/2 57.5%
*All*	23/9/0 71.9%	145/30/4 81.0%	40/34/0 54.1%	26/10 72.2%	61/21/0 74.4%	65/34/1 65.0%	43/16/1 71.7%	78/55/0 58.6%	89/66/0 57.4%	90/72/2 54.9%	25/22/5 48.1%	685/369/13 64.2%
Conduct disorder *												

*Boys*	8/19/0 29.6%	73/87/4 44.5%	8/61/0 11.6%	14/18/0 43.8%	21/55/0 27.6%	22/62/1 25.9%	5/47/0 9.6%	22/98/0 18.3%	34/101/0 25.2%	24/109/1 17.9%	5/32/6 11.6%	236/689/12 25.2%
*Girls*	2/3/0 40.0%	8/7/0 53.3%	0/5/0 0.0%	1/3/0 25.0%	2/4/0 33.3%	2/13/0 13.3%	2/5/1 25.0%	0/13/0 0.0%	3/17/0 15.0%	3/26/1 10.0%	1/8/0 11.1%	24/104/2 18.5%
*All*	10/22/0 31.3%	81/94/4 45.3%	8/66/0 10.8%	15/21/0 41.7%	23/59/0 28.0%	24/75/1 24.0%	7/52/1 11.7%	22/111/0 16.5%	37/118/0 22.4%	27/135/2 16.5%	6/40/0 11.5%	260/793/14 24.4%
Anxiety disorders *												

*Boys*	12/15/0 44.4%	78/80/6 47.6%	22/47/0 31.9%	12/20/0 37.5%	34/42/0 44.7%	25/58/2 29.4%	12/40/0 23.1%	43/77/0 35.8%	89/46/0 65.9%	53/80/1 39.6%	25/14/4 58.1%	405/519/13 43.2%
*Girls*	2/3/0 40.0%	12/3/0 80.0%	1/4/0 20.0%	1/3/0 25.0%	3/3/0 50.0%	4/11/0 26.7%	3/4/1 37.5%	7/6/0 53.8%	13/7/0 65.0%	16/13/1 53.3%	5/4/0 55.6%	67/61/2 51.5%
*All*	14/18/0 43.8%	90/83/6 50.3%	23/51/0 31.3%	13/23/0 36.1%	37/45/0 45.1%	29/69/2 29.0%	15/44/1 25.0%	50/83/0 37.6%	102/53/0 65.8%	69/93/2 42.1%	30/18/4 57.7%	472/580/15 44.2%
Mood disorders *												

*Boys*	3/24/0 11.1%	30/129/5 18.3%	4/65/0 5.8%	1/31/0 3.1%	6/70/0 7.9%	10/73/2 11.8%	5/47/0 9.6%	13/107/0 10.8%	45/90/0 33.3%	16/117/1 11.9%	3/36/4 7.0%	136/789/12 14.5%
*Girls*	1/4/0 20.0%	8/7/0 53.3%	0/5/5 0.0%	0/4/0 0.0%	1/5/0 16.7%	2/13/0 13.3%	2/5/1 25.0%	1/12/0 7.7%	6/14/0 30.0%	7/22/1 23.3%	0/9/0 0.0%	28/100/2 21.5%
*All*	4/28/0 12.5%	38/136/5 21.2%	4/70/0 5.4%	1/35/0 2.8%	7/75/0 8.5%	12/86/2 12.0%	7/52/1 11.7%	14/119/0 10.5%	51/104/0 32.9%	23/139/2 14.0%	3/45/4 5.8%	164/889/14 15.4%

* Numbers in cells indicate: "possible diagnosis"/"no diagnosis"/"n.a."; percentage of "possible diagnosis"

° For abbreviations see text

A hierarchical log-linear analysis of cell differences with regard to gender and comorbid condition revealed centre effects for all four conditions and a gender effect for mood disorders.

The final model for ODD retained all main effects and interactions. Partial associations were significant for ODD by centre, χ_p_^2^=51.8, df = 10, p < .001, but not for ODD by gender. ODD rates ranged from 48.1% up to 81.0% across centres. The final model for CD retained the gender effect and the centre by CD interaction effect. Partial associations were significant for CD by centre, χ_p_^2^=73.8, df = 10, p < .001, but not for CD by gender. CD rates in centres ranged from 10.8% to 45.3%. The final model for ANX retained the gender effect and the centre by ANX interaction effect. Partial associations were significant for ANX by centre, χ_p_^2^=66.4, SD = 10, p < .001, but not for ANX by gender. ANX rates in centres ranged from 25% to 65.8%. The final model for MOOD retained the two interaction effects of gender by MOOD and centre by MOOD. Partial associations were significant for MOOD by centre, χ_p_^2^=59.7, SD = 10, p < .001, and for MOOD by gender, χ_p_^2^=4.2, df = 1, p = .041, indicating that MOOD differed between centres (range 5.4% to 32.9%) and that a higher proportion of girls (21.5%) compared to boys (14.5%) were affected.

The frequencies of remaining conditions in the proband sample were assessed only at syndrome level and were clearly less prevalent. Obsessive compulsive disorder was possibly present in 35 boys (3.7%) and 4 girls (3.1%), Tourette's syndrome was possibly present in 22 boys (2.3%) and 3 girls (2.3%), substance abuse was possibly present in 19 boys (2%) and 1 girl (0.8%), psychosis was possibly present in 8 boys (0.9%) and 2 girls (1.5%), bipolar affective disorder was possibly present in 4 boys (0.4%) and 3 girls (2.3%), and reactive attachment disorder was possibly present in 4 boys (0.4%) and 1 girl (0.8%).

## Discussion

The present paper deals with the analysis of behavioural data of the International Multi-centre ADHD Genetics (IMAGE) project. The main focus is on the impact of the multi-centre design and the diagnostic procedure on the homogeneity of the data. Aggregating data from several recruiting centres is an important research strategy in order to enlarge sample sizes and, thus, to increase statistical power which is needed for generalising results, i.e., for achieving a needed level of significance.

The sample size is essential particularly in genetic linkage analyses of complex traits like ADHD when searching for markers contributing only to a small extent to the risk of ADHD [19]. While statistical power can be enlarged by increasing the sample size, it may be also reduced by factors influencing sample homogeneity by introducing uncontrolled or uncontrollable variance. The following discussion of the results will mainly focus on issues of sample homogeneity.

Despite the identical inclusion criteria (in terms of the numbers of ADHD symptoms) for children of all ages, we found a negative correlation between age and the mean number of hyperactive symptoms in the probands sample: older probands had lower numbers of symptoms than younger probands. At first sight, this could be interpreted as decreasing disease severity with age in our sample.

The interpretation of this age effect, however, must take into account the interplay between population characteristics and the diagnostic procedure. Previous studies have shown that age is an important factor moderating the symptoms of ADHD, resulting in a general symptom decline [8]. This is clearly underlined by normative sample data used in the IMAGE project, e.g., the CTRS [63]: a six year old girl having a score of thirteen on the CTRS DSM-IV hyperactivity scale deviates two standard deviations from the mean (T = 70), whereas a sixteen years old girl having the same score deviates twice as much, i.e. four standard deviations, from the mean (T = 90). Age effects in inattentive scores of the normative sample are less pronounced, but in the same direction. Thus, many adolescents probably had more hyperactive/impulsive or inattentive symptoms when they were younger.

These age effects have two important consequences in terms of disease severity: First, adolescents may deviate to a stronger extent from the normative mean in comparison to young children with the same number of ADHD symptoms present. Secondly, some evidence for genotype differences in groups differing in age but not in the number of ADHD symptoms can be derived. Therefore, if ADHD is seen as a quantitative trait, a probabilistic positive association may be assumed between the degree a phenotype (e.g. measured by dimensional questionnaire scores) deviates from the population mean and the number of alleles present, which are associated with the trait [19,64]. If a sample of individuals with an identical number of ADHD symptoms, but of different age, is virtually retraced to the age of five years, the mean number of ADHD symptoms in the older individuals would probably become smaller due to the negative correlation between age and symptoms in the population. In this virtual sample, as the quantitative trait hypothesis states, individuals with lower symptom numbers, i.e. a less deviating phenotype, have a lower probability of carrying an allele associated with the trait [64]. Because this is true for all susceptibility genes, it may be argued that individuals with fewer symptoms have a lower overall probability of carrying alleles associated with ADHD than individuals with more symptoms, irrespective of genetic interactions and environmental factors. As a final consequence, adolescents of our proband sample may have a higher genetic risk for ADHD than young probands with the same number of ADHD symptoms.

This implicit age effect inferred from epidemiological studies and a normative sample was moderated by the (small) negative correlation between age and the number of hyperactivity symptoms in our sample. As a consequence of these two features, one has to assume that, on the one hand, adolescents in our proband sample differed on average to a smaller extent from young children than inferred from the normative sample. On the other hand, individual differences between adolescents and young children with an identical number of symptoms remained. Therefore, we must conclude that the disease severity in terms of the deviation from normality increased with age, and that this effect was not represented in the number of ADHD symptoms present.

We found no age differences between boys and girls in the proband sample. But among the 11 centres there were ten significant pairwise differences in mean age ranging from 1.4 to 2.6 years, with effect sizes between 0.9 and 0.5. As a consequence, one would expect that centres with rather young probands, e.g. ESP_V, would have a relatively lower mean number of hyperactive symptoms, indicated by a lower rank after age correction, and vice versa (see Figure S2 in the Additional file 2). In fact, the change of rank position in only two centres (ESP_V and GER_E) was consistent with this hypothesis regarding the direction of rank change. However, the mean number of hyperactivity symptoms in these two centres was almost identical, so that the centre effects on hyperactive symptoms were moderated only marginally by age, probably at least partly due to the restricted range of symptom numbers in probands. Again, the effect of age differences between centres on hyperactive symptom number differences between centres was smaller than expected due to the moderating effect of declining hyperactive symptom numbers with age in our proband sample.

Gender was an additional source of heterogeneity with respect to ADHD symptoms and the comorbid conditions which are usually more frequent in boys [65]. Again, the normative sample underlying the DSM-IV scores of the CTRS [63] illustrates the differences attributable to gender: a T-score of 70 in the DSM-IV inattention scale is associated with a raw score of 16 in six year old boys, but with a raw score of only 8 in girls of the same age; in the DSM-IV hyperactivity/impulsivity scale the analogue scores are 18 in boys but only 10 in girls.

The proband sample of the present study had a homogeneous gender structure due to an absence of age differences between boys and girls and equal gender ratios across centres. Consequently, we can exclude that centre effects or gender effects on dependent variables were confounded by age effects.

The investigation of gender effects in the probands revealed no direct effects in most of the variables associated with the diagnostic procedure (i.e., the number of hyperactive symptoms, the age at inattention and hyperactivity detection, medication, all PACS ADHD symptoms, sixteen out of eighteen CPRS symptoms, and three of the four comprehensively assessed comorbidities, namely CD, ODD, and ANX). Exceptions were higher frequencies of inattentive symptoms (PACS and CTRS combined) in boys compared to girls and higher frequencies in boys for two thirds of the ADHD symptoms in the CTRS.

These differences were consistent with a meta-analysis reporting higher ADHD symptoms in boys compared to girls [65]. Concordant with gender differences in the normative sample, we conclude that girls in our proband sample deviated to a greater extent from normality than boys, even though the girls' symptom counts were similar or slightly lower than those of the boys - and that the use of equal symptom criteria in boys and girls introduced heterogeneity into the probands sample.

The multi-centre design is another possible source of sample heterogeneity. Analyses of the proband sample showed that centre effects played a more important role than age or gender effects. The centres differed significantly in age, in both inattentive and hyperactive/impulsive symptom numbers, in age of detection of both inattentive and hyperactive/impulsive symptoms, in fifteen out of eighteen ADHD symptoms in the PACS interview, in seventeen out of eighteen ADHD symptoms in the CPRS, in all ADHD symptoms in the CTRS, in five out of nine combined (PACS and CTRS) inattentive, and six out of nine combined hyperactive/impulsive symptoms, and in all comprehensively assessed comorbid conditions (CD, ODD, ANX, and MOOD).

Even if we would assume that centres did not differ with respect to genetic, socio-cultural, and methodological aspects, differences in gender and age ratios between centres, combined with differing sample sizes, could enhance the sample variance and introduce additional heterogeneity due to variables that were associated with gender or age. Such indirect effects were, however, either absent in the proband sample (gender), or only played a minor role with respect to ADHD symptoms (age), as discussed above. Consequently, we must assume that other factors caused the differences in psychopathology measures between centres (e.g. genotype differences [46], socio-cultural population differences, regional demographic factors, or specific health care structures leading to specific recruiting strategies). Not at least, different implicit normative backgrounds associated with sociocultural factors may have led to different ratings of objectively identical behaviour.

The hypothesis of a genotypic north-south factor [46] had no evident phenotypic equivalent in the probands with respect to symptom numbers, age of symptom detection, and frequencies of individual diagnostic symptoms. Centre differences in any of these variables did not build recognizable geographic patterns and neighbouring centres did not cluster more than distant centres did. Even national clusters were not recognisable to an extent that would justify dividing our sample into units of countries instead of centres. For example, there were some considerable and significant age differences (adjusted for multiple testing) between the two centres from both Germany and Israel, within-country-differences in the mean number of inattentive symptoms in Israel and the Netherlands, differences in the mean number of hyperactive symptoms (Netherlands, Israel, Germany), and also for age at symptom detection (Israel).

In summary, there were notable differences between centres in ADHD and comorbid symptoms. Although the variations of ADHD symptoms across centres remained within the diagnostic boundaries of ADHD-CT, we conclude that the significant centre differences result in a broader phenotypic range compared to a hypothetical sample of the same size with a single recruiting centre only. In particular with respect to genetic analyses and analyses of endophenotypes, power is, on the one hand, increased by expanding the sample size but, on the other hand, decreased by using a multi-centre recruiting strategy. Choosing a single-centre strategy, even if more time is needed for recruiting, is probably still the favourite strategy with respect to statistical power.

We investigated the differential influence of diagnostic symptoms in the diagnostic process. The higher discriminatory weights of hyperactive symptoms compared to inattentive symptoms in a binary logistic regression indicate that only few and predominantly hyperactive symptoms were needed to discriminate between probands and controls. Concordant with other findings, this result challenges the diagnostic system of the DSM-IV weighing all symptoms equally in an additive algorithm [66-68]. Interpretations going beyond this general statement, however, are neither appropriate nor intended due to methodological restrictions of the present study. For example, the 79 siblings of the control sample were part of those 339 siblings, who underwent the full diagnostic procedure due to suspected ADHD. Even if they did not reach the diagnostic threshold, many of them probably were subclinical cases, as indicated by a mean ADHD-symptom frequency of 51%, which corresponded to 9 positive symptoms out of 18 on average.

To investigate informant effects and instrument effects, we compared the two diagnostic sources PACS and CTRS and compared them to a third source, the CPRS, which was not implemented in the diagnostic procedure, but was used for screening. We found higher symptom frequencies in the parents' ratings compared to the teachers' ratings in the proband sample, independent of whether the CPRS or the PACS was compared to the CTRS. These findings are in accordance with known contrasts often seen in parent ratings that result from the direct comparison between two children and lead to a relative overestimation of the probands' symptoms compared to the siblings' symptoms [69,70]. In addition, medication may play a role, because some of the children were medicated continuously at school, but not at home.

Within the parents' ratings, the interview led to higher frequencies for 13/18 symptoms than the questionnaire. This higher sensitivity of the PACS for 2/3 of the symptoms may be a consequence of the more objective diagnostic conceptualization of the PACS, which assesses symptoms to a lesser extent by an implicit deviance rating, as the questionnaire does, but rather by asking how frequent and how intensive a symptom occurs. In contrast to this general tendency, the ability to sustain attention (IA2) is recorded much more frequently by the CPRS (84% in boys, 79% in girls) than by the PACS (46%, 42%). A comparison between symptoms and between the three diagnostic sources suggests that the sensitivity of the PACS is too weak for this symptom. The higher general sensitivity of the interview compared to the questionnaire, however, does not imply a lack in the utility of questionnaires as screening instruments. In general, the cut-off criteria of screening instruments were set far below the diagnostic threshold (we used a T-score of 63), so that all subjects, who reach the diagnostic threshold of the interview, but not of the questionnaire, were positively screened in all cases.

Informant and contrast effects were analysed and discussed on the basis of continuous data in the second part of this contribution, which concentrates on questionnaire scores [47].

Some further comments on the diagnostic procedure with respect to heterogeneity in the proband sample should be made. The diagnostic procedure of the IMAGE study used a teachers' questionnaire (CTRS) and a parental interview (PACS) in combination, by counting a symptom as present if it was present *either *in the CTRS *or *in the PACS. To prevent diagnoses based on a single informant only, at least two of the symptoms had to be present in both settings. This algorithm allows children to be positively diagnosed, even if their symptoms level is below the diagnostic threshold at home, at school, and even in both settings. This effect becomes evident when the frequencies of combined (PACS or CTRS) diagnostic symptoms (Table 3) is compared to the frequencies of each source alone (Figure 1). Whereas the most infrequent symptom occurs in about 80% of the probands when PACS and CTRS are combined, the lowest frequencies in PACS alone (<50%), CPRS alone (<60%), and CTRS alone (<40%) are clearly lower. The applied procedure may have excluded children without pervasive problems, but, on the other hand, also broadened the variety of symptom patterns in the proband sample and included some cases classified as subclinical in one or both settings. If we assume that some genetic variants interact differentially with the environmental conditions (home or school), the applied diagnostic algorithm classifying symptoms independently of their environmental condition and the type of informant may have introduced an uncontrolled variance in the phenotype.

Finally, some comments should be made concerning the sibling sample of the present study. The sample of 339 diagnosed siblings was heterogeneous in various ways. The subtypes differed across centres and between genders. There were, in particular, higher rates of ADHD-CT in boys than in girls. The mean number of both inattentive and hyperactive/impulsive symptoms differed between gender (higher frequencies in boys) and between centres. Differences in implementing the criteria for conducting the sibling interview (e.g. 'clinical suspicion of ADHD') may have introduced a bias leading to the large differences in subtype frequencies across centres. Additionally, differences in personnel resources, in combination with the declared purpose of the sibling interview (excluding ADHD cases in the sibling sample), may have introduced a centre bias. For these reasons, we do not further discuss the findings on the selected siblings but refer to the second part of this contribution dealing with analyses of the complete sample of 1446 siblings based on questionnaire data [47].

## Conclusion

The present IMAGE project used a multi-centre design in order to reach an acceptable power for detecting genetic variants involved in ADHD. The multi-centre design may have led to additional heterogeneity in the sample, as demonstrated by the present contribution. Additionally, a diagnostic procedure invariant to age, gender, and informant, as used in the IMAGE study, may have enhanced the heterogeneity in the proband sample. Our data do not allow us to define an optimal trade-off between sample size and sample homogeneity. In conclusion, we recommend that genetic analyses be either statistically adjusted for the known sources of variance (age, gender, centres) or that they be stratified by sources of heterogeneity. Removal of outliers or using robust statistics might also enhance statistical power.

## Abbreviations

DSM-IV criteria for ADHD: *IA1 *Often fails to give close attention to details or makes careless mistakes in schoolwork, work, or other activities; *IA2 *Often has difficulty sustaining attention in tasks or play activities; *IA3 *Often does not seem to listen when spoken to directly; *IA4 *Often does not follow through on instructions and fails to finish schoolwork, chores, or duties in the workplace (not due to oppositional behavior or failure to understand instructions); *IA5 *Often has difficulty organizing tasks and activities; *IA6 *Often avoids, dislikes, or is reluctant to engage in tasks that require sustained mental effort (such as schoolwork or homework); *IA7 *Often loses things necessary for tasks or activities (eg, toys, school assignments, pencils, books, or tools); *IA8 *Is often easily distracted by extraneous stimuli; *IA9 *Is often forgetful in daily activities; *HYP1 *Often fidgets with hands or feet or squirms in seat; *HYP2 *Often leaves seat in classroom or in other situations in which remaining seated is expected; *HYP3 *Often runs about or climbs excessively in situations in which it is inappropriate (in adolescents or adults, may be limited to subjective feelings of restlessness); *HYP4 *Often has difficulty playing or engaging in leisure activities quietly; *HYP5 *Is often 'on the go' or often acts as if 'driven by a motor'; *HYP6 *Often talks excessively; *IMP7 *Often blurts out answers before questions have been completed; *IMP8 *Often has difficulty awaiting turn; *IMP9 *Often interrupts or intrudes on others (eg, butts into conversations or games)

## Competing interests

PA has consulted with, received education grants from or spoken at sponsored meetings for Shire, Janssen-Cilag, Eli-Lilly and Flynn Pharma. JB has been in the past 3 years a consultant to/member of advisory board of/and/or speaker for Janssen Cilag BV, Eli Lilly, Bristol-Myer Squibb, Organon/Shering Plough, UCB, Shire, Medice, Servier, and Servier. TB served in an advisory or consultancy role for Bristol-Myers Squibb, Desitin, Lilly, Medice, Novartis, Pfizer, Shire, UCB and Viforpharma. He received conference attendance support or received speaker's fee by Lilly, Janssen McNeil, Medice, Novartis, Shire, UCB. He received unrestricted grants for organizing a CME conference by Lilly, Janssen McNeil, Medice, Novartis, Shire, UCB. He is/has been involved in clinical trials conducted by Lilly, Shire and Novartis. The present work is unrelated to the above grants and relationships. SVF has, in the past year received consulting fees and has been on Advisory Boards for Eli Lilly, Ortho-McNeil and Shire Development and has received research support from Eli Lilly, Pfizer, Shire and the National Institutes of Health. In previous years, SVF has received consulting fees or has been on Advisory Boards or has been a speaker for the following sources: Shire, McNeil, Janssen, Novartis, Pfizer and Eli Lilly. In previous years he has received research support from Eli Lilly, Shire, Pfizer and the National Institutes of Health. RDO received support from Janssen and UCB during this period. HR has served as an advisor to Shire and received research support from Shire and Lilly and conference attendance support from Lilly.

The present study is unrelated to these relationships. AR declares the following competing interests: Advisory Board and Speakers Bureau: Lilly, Shire, Medice, Novartis; Research Support: Shire, German Research Society, Schwaabe; Travel Support: Shire; Educational Grant: Shire. JS declares the following competing interests: Advisory Board: Lilly, Shire, Research Grant(s) Lilly, Speaker's Fee: Shire, Lilly, Janssen-Cillag. ESB declares the following competing interests: Recent speaker board: Shire, UCB Pharma. Current & recent consultancy: UCB Pharma, Shire. Current & recent research support: Janssen Cilag, Shire, Qbtech, Flynn Pharma. Advisory Board: Shire, Flynn Pharma, UCB Pharma, Astra Zeneca. Conference support: Shire. HCS has served as an advisor and/or speaker to the following companies: Janssen-Cilag, Eli Lilly, Medice, Novartis, Shire, and UCB. MT has served as an advisor, speaker and had research grants from the following companies: Janssen-Cilag, Eli Lilly, Shire, and UCB. All other authors declare no competing interests to disclose.

## Authors' contributions

UCM and HCS jointly planned the analyses and drafted the manuscript with UCM performing all the statistical analyses. All other authors were principle investigators at the various centres and SVF was overall principle investigator of the IMAGE study. All authors commented on the manuscript and approved the final draft.

## Pre-publication history

The pre-publication history for this paper can be accessed here:

http://www.biomedcentral.com/1471-244X/11/54/prepub

## Supplementary Material

Additional file 1 **Figure S1**. Mean age of centre subsamples in ascending order, and significant post hoc pairwise comparisons. Click here for file

Additional file 2 **Figure S2**. Trimmed means of symptom numbers and age at symptom onset in probands (N = 1068). Click here for file

Additional file 3 **Table S1**. Diagnostic subtypes in the siblings sample. Click here for file

Additional file 4 **Figure S3**. Subtype frequencies in the siblings sample across centres and gender. Click here for file
