# Supplementary material for: The impact of study design and diagnostic approach in a large multi-centre ADHD study. Part 1: ADHD symptom patterns
Source: BMC Psychiatry. 2011 Apr 7;11:54. doi: 10.1186/1471-244X-11-54 (PMC3082291; doi:10.1186/1471-244X-11-54)
Supplement: Additional file 1 — Figure S1. Mean age of centre subsamples in ascending order, and significant post hoc pairwise comparisons. [file 1471-244X-11-54-S1.PDF]

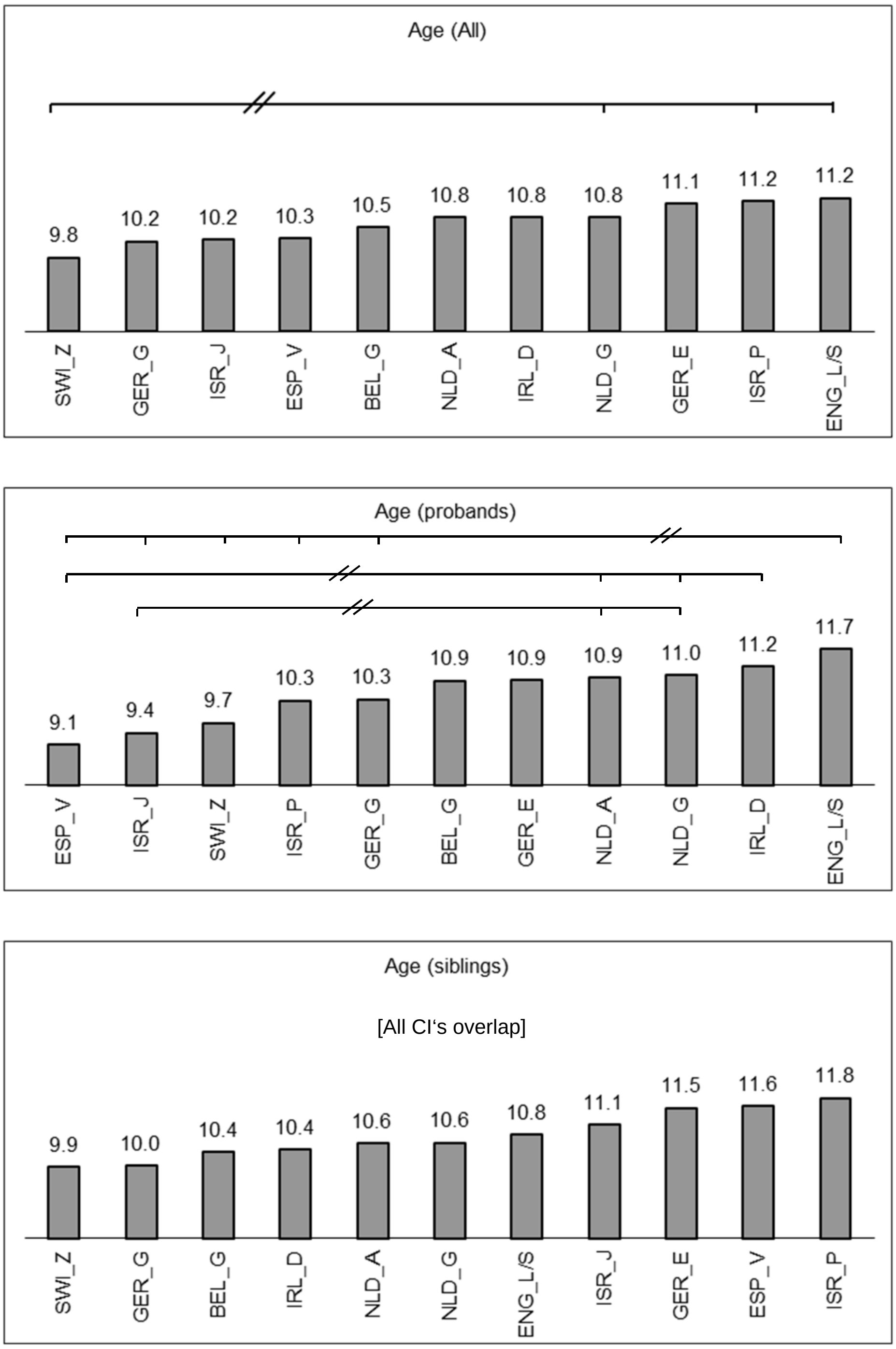

Figure S1
Mean age of centre subsamples in ascending order, and significant post hoc pairwise comparisons.
Notes: Horizontal lines with vertical marks indicate groups of significant pairwise comparisons; withing each group, the 95% familywise confidence interval (CI) of each centre indicated by a mark on the left of the double slash (//) had no overlap with the CI of each centre indicated by the mark on the right side of the double slash.
