# Supplementary material for: The impact of study design and diagnostic approach in a large multi-centre ADHD study. Part 1: ADHD symptom patterns
Source: BMC Psychiatry. 2011 Apr 7;11:54. doi: 10.1186/1471-244X-11-54 (PMC3082291; doi:10.1186/1471-244X-11-54)
Supplement: Additional file 2 — Figure S2. Trimmed means of symptom numbers and age at symptom onset in probands (N = 1068). [file 1471-244X-11-54-S2.PDF]

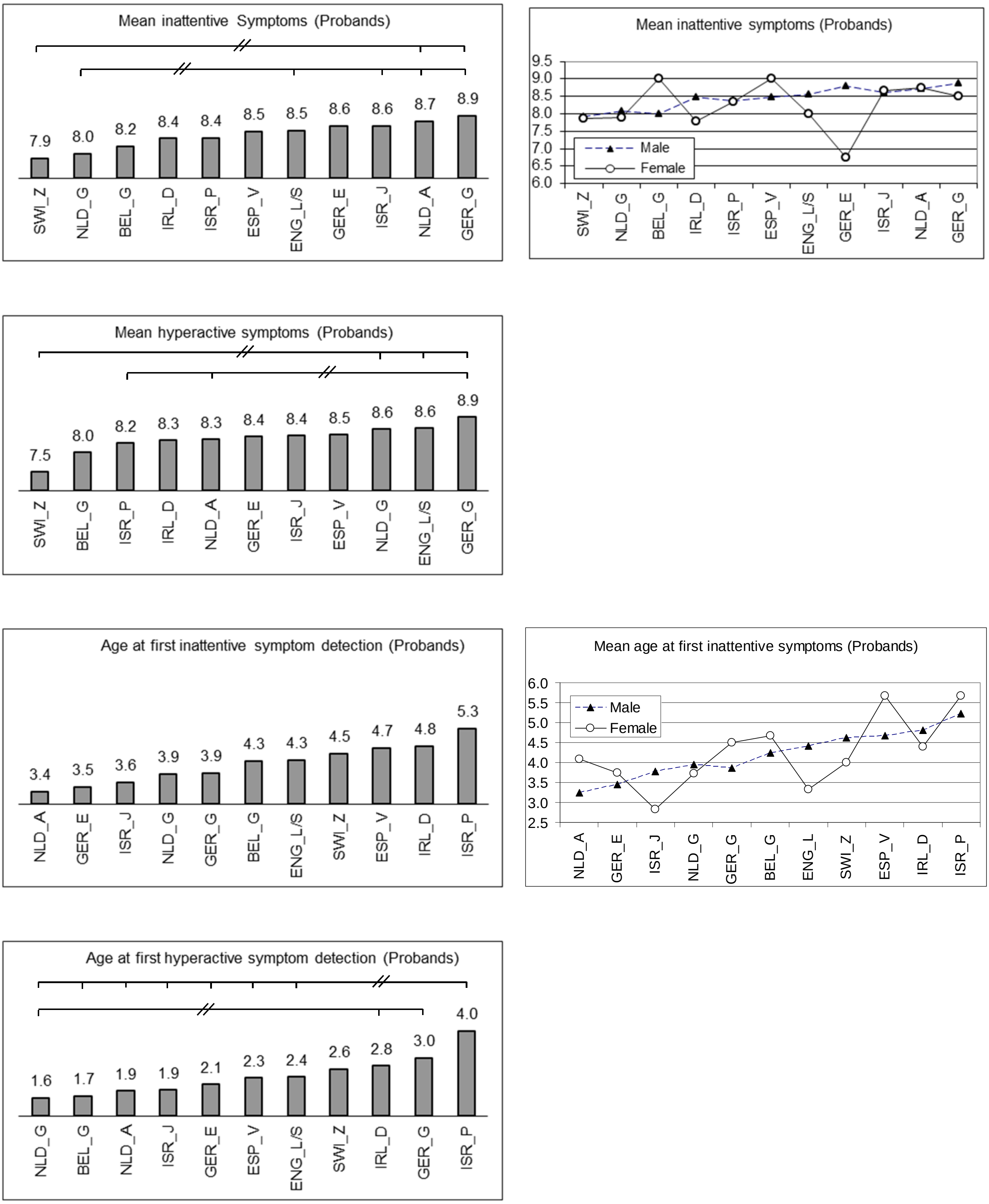

Figure S2

Trimmed means of symptom numbers and age at symptom onset in probands (N = 1068).

Notes: Figures on the left side show 20% trimmed means per centre in ascending order. Horizontal lines above the bars indicate subgroups of significant pairwise differences. Each centre indicated by a vertical mark at the left side of the double slash (//) significantly differs from each site indicated by a vertical mark on the right side of the double slash, defined as non-overlapping 95% familywise confidence intervals.

For each variable with a significant site by gender interaction, trimmed means in boys and girls are shown in the figures on the right side.
