# Supplementary material for: The impact of study design and diagnostic approach in a large multi-centre ADHD study. Part 1: ADHD symptom patterns
Source: BMC Psychiatry. 2011 Apr 7;11:54. doi: 10.1186/1471-244X-11-54 (PMC3082291; doi:10.1186/1471-244X-11-54)
Supplement: Additional file 3 — Table S1. Diagnostic subtypes in the siblings sample. [file 1471-244X-11-54-S3.PDF]

Table S1: Diagnostic subtypes in the siblings sample

	PACS Interview assessed															*PACS not assessed **		
	Combined type °			*Hyperactive type °*			*Inattentive type °*			*No diagnosis °*			*All Diagnoses °*					
	Boys	*Girls*	*All*	*Boys*	*Girls*	*All*	*Boys*	*Girls*	*All*	*Boys*	*Girls*	*All*	*Boys*	*Girls*	*All*	*Boys*	*Girls*	*All*
	8	-	8	-	-	-	5	1	6	3	-	3	16	1	17	20	12	32
BEL_G	100%	-	100%	-	-	-	83%	17%	100%	100%	-	100%				63%	38%	100%
	47%	-	47%	-	-	-	29%	6%	35%	18%	-	18%	94%	6%	100%	41%	24%	65%
	15	7	22	5	4	9	4	1	5	7	7	14	31	19	50	91	111	202
ENG_L/S	68%	32%	100%	56%	44%	100%	80%	20%	100%	50%	50%	100%				45%	55%	100%
	30%	14%	44%	10%	8%	18%	8%	2%	10%	14%	14%	28%	62%	38%	100%	36%	44%	80%
	4	1	5	-	-	-	-	-	-	-	-	-	4	1	5	36	34	70
ESP_V	80%	20%	100%	-	-	-	-	-	-	-	-	-				51%	49%	100%
	80%	20%	100%	-	-	-	-	-	-	-	-	-	80%	20%	100%	48%	45%	93%
	2	-	2	1	1	2	1	-	1	-	-	-	4	1	5	19	25	44
GER_E	100%	-	100%	50%	50%	100%	100%	-	100%	-	-	-				43%	57%	100%
	40%	-	40%	20%	20%	40%	20%	-	20%	-	-	-	80%	20%	100%	39%	51%	90%
	14	3	17	1	-	1	-	-	-	3	3	6	18	6	24	36	50	86
GER_G	82%	18%	100%	100%	-	100%	-	-	-	50%	50%	100%				42%	58%	100%
	58%	13%	71%	4%	-	4%	-	-	-	13%	13%	25%	75%	25%	100%	33%	45%	78%
	9	3	12	3	-	3	6	4	10	3	7	10	21	14	35	49	59	108
IRL_D	75%	25%	100%	100%	-	100%	60%	40%	100%	30%	70%	100%				45%	55%	100%
	26%	9%	34%	9%	-	9%	17%	11%	29%	9%	20%	29%	60%	40%	100%	34%	41%	76%
	5	1	6	-	-	-	-	1	1	-	1	1	5	3	8	22	37	59
ISR_J	83%	17%	100%	-	-	-	-	100%	100%	-	100%	100%				37%	63%	100%
	63%	13%	75%	-	-	-	-	13%	13%	-	13%	13%	63%	38%	100%	33%	55%	88%
	14	5	19	2	-	2	9	7	16	11	8	19	36	20	56	73	67	140
ISR_P	74%	26%	100%	100%	-	100%	56%	44%	100%	58%	42%	100%				52%	48%	100%
	25%	9%	34%	4%	-	4%	16%	13%	29%	20%	14%	34%	64%	36%	100%	37%	34%	71%
	25	8	33	1	4	5	15	10	25	5	8	13	46	30	76	60	79	139
NLD_A	76%	24%	100%	20%	80%	100%	60%	40%	100%	38%	62%	100%				43%	57%	100%
	33%	11%	43%	1%	5%	7%	20%	13%	33%	7%	11%	17%	61%	39%	100%	28%	37%	65%
	19	11	30	1	1	2	3	5	8	5	4	9	28	21	49	88	93	181
NLD_G	63%	37%	100%	50%	50%	100%	38%	63%	100%	56%	44%	100%				49%	51%	100%
	39%	22%	61%	2%	2%	4%	6%	10%	16%	10%	8%	18%	57%	43%	100%	38%	40%	79%
	3	1	4	1	1	2	-	4	4	2	2	4	6	8	14	21	25	46
SWI_Z	75%	25%	100%	50%	50%	100%	-	100%	100%	-	50%	100%				46%	54%	100%
	21%	7%	29%	7%	7%	14%	-	29%	29%	-	14%	29%	43%	57%	100%	35%	42%	77%
	118	40	158	15	11	26	43	33	76	39	40	79	215	124	339	515	592	1107
Total	35%	12%	47%	4%	3%	8%	13%	10%	22%	12%	12%	23%				47%	53%	100%
	75%	25%	100%	58%	42%	100%	57%	43%	100%	49%	51%	100%	63%	37%	100%	36%	41%	77%

° N / percentage within subtype / percentage within assessed siblings

* N / percentage within not assessed siblings / percentage within total siblings
