# Supplementary material for: The impact of study design and diagnostic approach in a large multi-centre ADHD study. Part 1: ADHD symptom patterns
Source: BMC Psychiatry. 2011 Apr 7;11:54. doi: 10.1186/1471-244X-11-54 (PMC3082291; doi:10.1186/1471-244X-11-54)
Supplement: Additional file 4 — Figure S3. Subtype frequencies in the siblings sample across centres and gender. [file 1471-244X-11-54-S4.PDF]

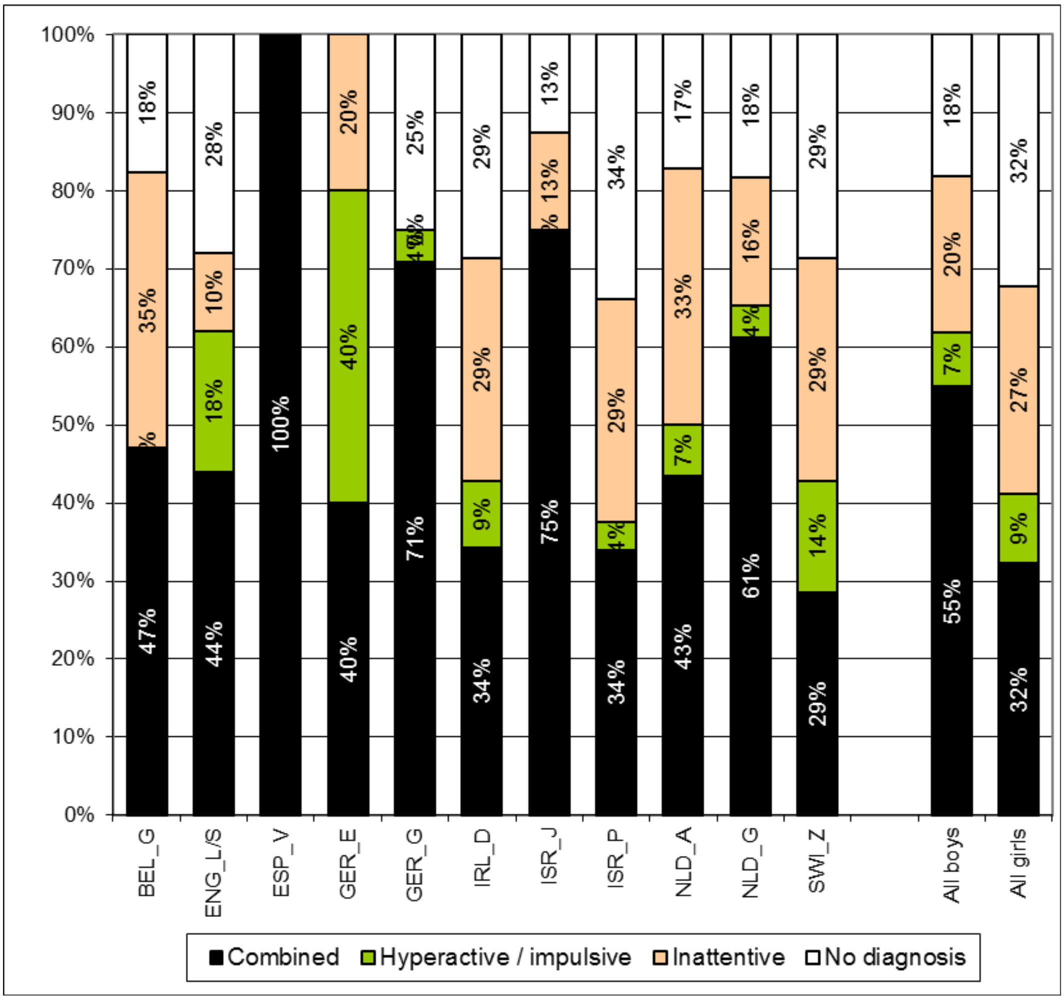

Figure S3

Subtype frequencies in the siblings sample across centres and gender.

Notes: 339 diagnosed siblings are included in the analysis. Exact numbers are provided in the additional file 3 (Table A1).
